# Supplementary material for: Rare variants and survival of patients with idiopathic pulmonary fibrosis
Source: medRxiv. 2024 Oct 15:2024.10.12.24315151. Preprint. [Version 1] doi: 10.1101/2024.10.12.24315151 (PMC11527076; doi:10.1101/2024.10.12.24315151)
Supplement: Supplement 1 [file NIHPP2024.10.12.24315151v1-supplement-1.pdf]

medRxiv preprint doi: https://doi.org/10.1101/2024.10.12.24315151; this version posted October 15, 2024. The copyright holder for this preprint (which was not certified by peer review) is the author/funder, who has granted medRxiv a license to display the preprint in perpetuity.
It is made available under a CC-BY 4.0 International license .

Supplementary material

Rare variants and survival of patients with idiopathic pulmonary fibrosis

Aitana Alonso-Gonzalez[1], David Jáspez[2], José M. Lorenzo-Salazar[2], Shwu-Fan Ma[3], Emma Strickland[3], Josyf Mychaleckyj[4], John S. Kim[3], Yong Huang[3], Ayodeji Adegunsoye[5], Justin M. Oldham[6], Philip L. Molyneaux[7,8], Toby Maher[7,8,9], Louise V Wain[10,11], Richard Allen[10], Martin D. Tobin[10,11], Jonathan Kropski[12], Brian Yaspan[13], Timothy S. Blackwell[12], David Zhang[14], Christine Kim Garcia[14,15], Fernando J. Martinez[16], Imre Noth[3], and Carlos Flores[1,2,17,18]

medRxiv preprint doi: https://doi.org/10.1101/2024.10.12.24315151; this version posted October 15, 2024. The copyright holder for this preprint (which was not certified by peer review) is the author/funder, who has granted medRxiv a license to display the preprint in perpetuity.
It is made available under a CC-BY 4.0 International license .

Supplementary methods 4
Description of study cohorts 4
Supplementary bioinformatics methods 4
Supplementary results 5
Prevalence of qualifying variants (QV) in PROFILE 5
Supplementary tables 6
Supplementary Table 1. Baseline characteristics and outcomes of IPF patients from stage one and stage two cohorts. 6
Supplementary Table 2. Regions of interest (ROIs) for qualifying variants annotations in hg38. .. 7
Supplementary Table 3. Alternative definitions for qualifying variants and the rare synonymous used for sensitivity analyses. 8
Supplementary Table 4. Common IPF risk variants and effects considered for PRS-IPF estimation. 9
Supplementary Table 5. Common telomere length variants and effects considered for PRS-TL estimation. 10
Supplementary Figures 11
Supplementary Figure 1. Principal component analysis.. 11
Supplementary Figure 2. Distribution of qualifying variants (QV) in monogenic adult-onset pulmonary fibrosis (PF) genes in the PFFPF and PROFILE cohorts. 12
Supplementary Figure 3. Association between prevalence of qualifying variants (QV) and PRS-IPF in the PFFPR. 13
Supplementary Figure 4. Association between prevalence of qualifying variants (QV) and PRS-IPF (after excluding the MUC5B locus) in the PFFPR. 14
Supplementary Figure 5. Association between the prevalence of qualifying variants (QV) and PRS-TL in the PFFPR. 15
Supplementary Figure 6. Association between prevalence of qualifying variants (QV) in telomere and non-telomere genes and PRS-TL in the PFFPR. 16
Supplementary Figure 7. Association between prevalence of qualifying variants (QV) in telomere genes and PRS-TL in the PFFPR. 17
Supplementary Figure 8. Kaplan-Meier survival analysis for qualifying variants (QV) (per gene and group of genes) and the MUC5B risk allele in the PFFPR. p-values for the log-rank test are shown. 18
Supplementary Figure 9. Qualifying variants (QV) effect on survival in the PFFPR (excluding carriers of QV within PARN). 19
Supplementary Figure 10. Alternative qualifying variants (QV) classifications and effects on survival in the PFFPR. 20
Supplementary Figure 11. Association between PRS-TL tertiles and survival in the PFFPR.. .. 21
Supplementary Figure 12. Association between high and low PRS-TL and survival in the PFFPR.. 22
Supplementary Figure 13. Association of PRS-IPF (after excluding the MUC5B locus) and survival in the PFFPR. Kaplan-Meier analysis showing p-values for the log-rank test. 23
Supplementary Figure 14. Associations between PRS-IPF and MUC5B rs35705950 genotypes with survival among carriers and non-carriers of qualifying variants (QV) in the PFFPR.. 24

medRxiv preprint doi: https://doi.org/10.1101/2024.10.12.24315151; this version posted October 15, 2024. The copyright holder for this preprint (which was not certified by peer review) is the author/funder, who has granted medRxiv a license to display the preprint in perpetuity.
It is made available under a CC-BY 4.0 International license .

Supplementary Figure 15. Association between prevalence of qualifying variants (QV) and PRS-IPF in PROFILE. 25
Supplementary Figure 16. **Kaplan-Meier survival analysis for qualifying variants (QV) (per gene and group PF genes) and the MUC5B risk allele in PROFILE. p-values for the log-rank test are shown.** 26

Supplementary references **27**

medRxiv preprint doi: https://doi.org/10.1101/2024.10.12.24315151; this version posted October 15, 2024. The copyright holder for this preprint (which was not certified by peer review) is the author/funder, who has granted medRxiv a license to display the preprint in perpetuity.
It is made available under a CC-BY 4.0 International license .

Supplementary methods

Description of study cohorts

The Pulmonary Fibrosis Foundation Patient Registry (PFFPR) is a large multicentre based registry that collects baseline and longitudinal demographic and clinical information about well-characterized patients with interstitial lung diseases (ILD) in the United States since March 2016 to allow retrospective and prospective research[1]. In addition, the PFFPR major objective is to apply blood-based omics technologies (whole-genome sequencing [WGS], proteomic analysis, and transcriptional profiling) on blood samples from patients to study molecular markers of the onset or progression of diseases. Patients aged ≥18 years old who has ILD diagnosed and had not undergone lung transplantation were recruited from approximately 42 USA sites selected primarily from the familial pulmonary fibrosis (FPF) Care Center Network. They were followed for the progression of the disease through the lifetime of the PFFPR or the patient until the patient receives lung transplant. More details of the PFFPR including inclusion and exclusion criteria as well as collected clinical variables are described elsewhere[1]. The PFFPR cohort includes 1317 individuals with ILD for whom WGS data are available. For this study, we included the 917 PFFPR patients with a definitive IPF diagnosis. Family history was available for all of them although no genetic causes were previously assessed. After the quality control procedures, 888 of those patients remained in the study **(Figure 1)**.

The PROFILE is a UK large, prospective, multicentre, longitudinal study conducted on patients with fibrotic ILD[2,3]. The cohort includes 541 patients with IPF or idiopathic non-specific interstitial pneumonia aged 18-85 recruited from tertiary specialist ILD and from local secondary care hospitals. Blood samples for genomic analysis were collected and they were followed for disease progression through 3 years. After quality control steps, the second stage of the study included 472 patients with a confirmed diagnosis of IPF **(Figure 1).**

Baseline characteristics of the PFFPR and the PROFILE cohorts are listed in **Supplementary Table 1**.

Supplementary bioinformatics methods

In both cohorts, several quality control (QC) analyses were performed: (i) detection of QC outliers, (ii) the kinship between patients, (iii) sample cross-contamination, and (iv) sex discordance. We used a combination of DRAGEN metrics, and assessments with PLINK v1.90b6.24[4], SCE-VCF v0.1.2 (https://github.com/HTGenomeAnalysisUnit/SCE-VCF), Somalier v0.2.19[5], and KING v2.3.2[6].

Detection of QC outliers: Based on PLINK analysis, we detected abnormal heterozygosity rate and genotyping call rate to infer potential sample contaminations and/or a low DNA concentration. A heterozygosity rate value ± 3 standard deviations from the mean and/or genotype call rate <0.95 were considered as outliers.

Kinship between patients: We detected duplicates or monozygotic twins, and first-degree kinship relationships with three different tools: we considered two samples as duplicates or obtained from monozygotic twins if a PI_HAT value was >0.9 for PLINK, a Somalier relatedness value >0.9, and a KING kinship coefficient >0.354. We considered as first-degree relatives a PI_HAT in the range of 0.4-0.6 for PLINK, a Somalier relatedness value in the range of 0.4-0.6, and a KING kinship coefficient in the range of 0.177-0.354. We found a complete consensus among these tools in the cohort. Second-degree relatives were not detected.

medRxiv preprint doi: https://doi.org/10.1101/2024.10.12.24315151; this version posted October 15, 2024. The copyright holder for this preprint (which was not certified by peer review) is the author/funder, who has granted medRxiv a license to display the preprint in perpetuity.
It is made available under a CC-BY 4.0 International license .

Sample cross-contamination: We used the "*estimated_sample_contamination*" parameter from DRAGEN metrics to exclude samples with evidence ≥2% of contamination. We also used SCE-VCF tool, which estimates contamination from VCF files using the CHARR method[7], based on the recommended thresholds to consider a sample as contaminated (CHARR > 0.03 and INCONSISTENT_AB_HET_RATE > 0.15). We found a complete consensus among these tools in the identification of potential sample contamination in the PFF-PR. For PROFILE, we only relied on SCE-VCF for the sample cross-contamination inference.

Sex discordance: Biological sex inference from genetic data was obtained with Somalier following recommendations. For that we compared the scaled mean depth on X and Y chromosomes for 365 and 17 genomic positions, respectively. Sex discordance, identified by comparing the genetically inferred sex with that recorded, was also used to exclude patients from the study. In the PFF-PR, a female was identified as a possible X0 aneuploid due to the low number of heterozygous sites on the X chromosome and was excluded from the analysis.

Supplementary results

Prevalence of qualifying variants (QV) in PROFILE

The genes with the highest burden of QVs were: *RTEL1* (20.5%), *TERT* (15.1%), and *PARN* (17.8%) **(Supplementary Figure 2B**, **2D)**. The prevalence of QVs among carriers of the risk *MUC5B* genotype (rs35705950-T) was lower (14.97%) than among those carrying the protective GG genotype (16.85%), although the difference was not statistically significant (*p*=0.60). We observed the same effect direction as in PFFPR when assessing the association between the lower PRS-IPF tertile and reduced survival (HR=1.49, 95% CI=0.14-1.95, $p=3.1\text{X}10^{-3}$) **(Figure 3B)**.

medRxiv preprint doi: https://doi.org/10.1101/2024.10.12.24315151; this version posted October 15, 2024. The copyright holder for this preprint (which was not certified by peer review) is the author/funder, who has granted medRxiv a license to display the preprint in perpetuity.
It is made available under a CC-BY 4.0 International license .

Supplementary tables

Supplementary Table 1. Baseline characteristics and outcomes of IPF patients from stage one and stage two cohorts.

Characteristics	PFFPR (n=888)*	PROFILE (n=472)$
Age, yr, mean (SD)	71.02 (7.8)	70.65 (7.9)
Male, n (%)	676 (76.1%)	366 (77.5%)
Ethnicity, n (%)		
Unknown	17	
Asian	23	-
Black	10	
White	838	
Ever smoker, n, (%)	571 (64.3%)	326 (69.1%)
Familial history, n, (%)	176 (19.8 %)	-
FVC% predicted, mean (SD)	67.74 (16.79)	78.97 (19.01)
DLCO% predicted, mean (SD)	29.3 (4.84)	44.97 (14.98)
Dead, n, (%)	337 (37.9%)	346 (73.3%)
Transplant, n (%)	139 (15.6%)	-
Mean survival in years (IQR)	4.86 (3.31-6.93)	3.03 (1.7-5.71)
MUC5B genotype with risk allele	622 (70%)	294 (62.3%)

Abbreviations: PFFPR, The Pulmonary Fibrosis Foundation Patient Registry; SD, standard deviation; FVC, Forced vital capacity; DLCO, predicted diffusing capacity of the lungs for monoxide; IQR=interquartile range. *Missing data: FVC predicted (n=41) and DLCO predicted (n=68); $Missing data: FVC predicted (n=12) and DLCO predicted (n=50).

medRxiv preprint doi: https://doi.org/10.1101/2024.10.12.24315151; this version posted October 15, 2024. The copyright holder for this preprint (which was not certified by peer review) is the author/funder, who has granted medRxiv a license to display the preprint in perpetuity.
It is made available under a CC-BY 4.0 International license .

Supplementary Table 2. Regions of interest (ROIs) for qualifying variants annotations in hg38.

Chromosome	Gene	Start	End
5	*TERT*	1,253,047	1,295,168
3	*TERC*	169,764,420	169,765,160
14	*TINF2*	24,238,186	24,242,763
X	*DKC1*	154,762,642	154,777,789
20	*RTEL1*	63,657,710	63,696,353
16	*PARN*	14,435,600	14,632,828
4	*NAF1*	163,109,973	163,166,990
12	*ZCCHC8*	122,471,500	122,501,032
8	*SFTPC*	22,156,813	22,164,579
10	*SFTPA2*	79,555,752	79,560,507
10	*SFTPA1*	79,610,839	79,615,555
5	*SPDL1*	169,583,536	169,604,878
3	*KIF15*	44,761,621	44,873,476

medRxiv preprint doi: https://doi.org/10.1101/2024.10.12.24315151; this version posted October 15, 2024. The copyright holder for this preprint (which was not certified by peer review) is the author/funder, who has granted medRxiv a license to display the preprint in perpetuity.
It is made available under a CC-BY 4.0 International license .

Supplementary Table 3. Alternative definitions for qualifying variants and the rare synonymous used for sensitivity analyses.

	Ultra-rare PTV	Ultra-rare Ensemble# (PTV + Missense + Indel)	Rare PTV only	Rare Ensemble# (PTV + Missense + Indel)	Semi-rare PTV only	Semi-rare Ensemble# (PTV + Missense + Indel)	Rare synonymous^
Missense AF*	-	0	-	0.0005	-	0.01	-
PTV AF*	0	0	0.001	0.001	0.01	0.01	-
Consensus in silico prediction for missense:&							
Polyphen2 Humdiv	-	Probably	-	Probably	-	Probably	-
REVEL	-	>0.5	-	>0.5	-	>0.5	-
PrimateAI	-	>0.8	-	>0.8	-	>0.8	-
Variants (n)	13	30	28	67	29	78	38

*Below threshold for any population in gnomAD v2.1 exomes (AFR, AMR, ASJ, EAS, FIN, NFE, OTH, SAS) or gnomAD v3.2 genomes (AFR, AMR, ASJ, EAS, FIN, MID, NFE, OTH, SAS) or in The 1000 Genomes Project Phase 3 genomes (AFR, AMR, EAS, EUR, SAS).

&Consensus of three predictors (Polyphen2, REVEL, PrimateAI) for missense variants only if >2 out of 3, or 2 out of 2, or 1 out of 1 filters pass. Some predictors may have missing values.

^Allele frequency cutoff of 0.0005 in any population in gnomAD v2.1 exomes (AFR, AMR, ASJ, EAS, FIN, NFE, OTH, SAS) or gnomAD v3.2 genomes (AFR, AMR, ASJ, EAS, FIN, MID, NFE, OTH, SAS) or in The 1000 Genomes Project Phase 3 genomes (AFR, AMR, EAS, EUR, SAS), only synonymous variants.

#Ensemble models include non-coding *TERC* variants selected if passing missense AF level and involved in intramolecular base-pairing or previously described in pulmonary fibrosis or dyskeratosis congenita or hoyeraal hreidarsson.

PTV: Protein truncating variants.

medRxiv preprint doi: https://doi.org/10.1101/2024.10.12.24315151; this version posted October 15, 2024. The copyright holder for this preprint (which was not certified by peer review) is the author/funder, who has granted medRxiv a license to display the preprint in perpetuity.
It is made available under a CC-BY 4.0 International license .

Supplementary Table 4. Common IPF risk variants and effects considered for PRS-IPF estimation.

Locus	SNP ID	Chr.	POSITION (hg38)	EFFECT	NON EFFECT	OR	P
KIF15	rs141979279	3	44,816,639	C	T	1.50	$1.21x10^{-10}$
TERC	rs10936601	3	169,810,661	C	T	0.79	$2.10x10^{-15}$
FAM13A	rs2013701	4	88,963,935	G	T	1.25	$4.60x10^{-16}$
TERT	rs7725218	5	1,282,299	G	A	1.41	$4.90x10^{-32}$
DSP	rs2076295	6	7,562,999	G	T	1.49	$1.50x10^{-48}$
MAD1L1	rs12699415	7	1,869,843	A	G	1.27	$7.85x10^{-18}$
ZKSCAN1	rs2897075	7	100,032,719	T	C	1.30	$1.77x10^{-21}$
DEPTOR	rs28513081	8	119,921,886	A	G	1.20	$1.22x10^{-9}$
10q25.1	rs79684490	10	109,470,103	A	G	1.40	$3.52x10^{-8}$
MUC5B	rs35705950	11	1,219,991	T	G	5.06	$9.09x10^{-418}$
ATP11A	rs12585036	13	112,881,427	C	T	1.29	$5.99x10^{-14}$
IVD	rs59424629	15	40,428,343	T	G	1.27	$4.98x10^{-19}$
KNL1	rs12912339	15	40,639,510	A	G	1.30	$7.41x10^{-13}$
AKAP13	rs62023891	15	85,553,985	A	G	1.18	$1.32x10^{-8}$
NPRL3	rs74614704	16	112,241	A	G	1.49	$2.57x10^{-12}$
17q21.31	rs3785884	17	45,980,229	G	A	1.40	$2.53x10^{-20}$
DPP9	rs35574495	19	4,686,976	G	T	0.80	$1.08x10^{-9}$
STMN3	rs112087793	20	63,652,817	C	T	1.34	$1.09x10^{-8}$
RTEL1	rs41308092	20	63,693,038	A	G	1.75	$3.13x10^{-9}$

SNP: Single nucleotide polymorphism; Chr.: chromosome; OR: odds ratio; P: significance in the original study

medRxiv preprint doi: https://doi.org/10.1101/2024.10.12.24315151; this version posted October 15, 2024. The copyright holder for this preprint (which was not certified by peer review) is the author/funder, who has granted medRxiv a license to display the preprint in perpetuity.
It is made available under a CC-BY 4.0 International license .

Supplementary Table 5. Common telomere length variants and effects considered for PRS-TL estimation.

Locus	SNP ID	CHR	POSITION (hg38)	EFFECT	NON EFFECT	BETA	P
PARP1	rs3219104	1	226374920	C	A	0.042	9.60×10^{-11}
TERC	rs10936600	3	169796797	T	A	-0.086	7.18×10^{-51}
NAF1	rs4691895	4	163127047	C	G	0.058	1.58×10^{-21}
TERT	rs7705526	5	1285859	A	C	0.082	5.34×10^{-45}
TERT	rs2853677	5	1287079	A	G	-0.064	3.35×10^{-31}
POT1	rs59294613	7	124914213	A	C	-0.041	1.17×10^{-13}
STN1	rs9419958	10	103916188	C	T	-0.064	5.05×10^{-19}
ATM	rs228595	11	108234866	A	G	-0.029	1.43×10^{-8}
DCAF4	rs2302588	14	72938044	C	G	0.048	1.68×10^{-8}
MPHOSPH6	rs7194734	16	82166375	T	C	-0.037	6.94×10^{-10}
ZNF208	rs8105767	19	22032639	G	A	0.039	5.42×10^{-13}
RTEL1/ STMN3	rs75691080	20	63638397	T	C	-0.067	5.99×10^{-14}
RTEL1	rs34978822	20	63660246	G	C	-0.140	7.26×10^{-10}
RTEL1/ ZBTB46	rs73624724	20	63805045	C	T	0.051	6.33×10^{-12}
SENP7	Rs551442	3	101346524	T	C	-0.037	2.45×10^{-8}
MOB1B	rs13137667	4	70908630	C	T	0.077	2.43×10^{-8}
CARMIL1	rs34991172	6	25480100	G	T	-0.061	6.19×10^{-9}
PRRC2A	rs2736176	6	31619784	C	G	0.035	3.53×10^{-10}
TERF2	rs3785074	16	69373083	G	A	0.035	4.64×10^{-10}
RFWD3	rs62053580	16	74646176	G	A	-0.039	4.08×10^{-8}

SNP: Single nucleotide polymorphism; CHR: chromosome; OR: odds ratio; P: significance in the original study

medRxiv preprint doi: https://doi.org/10.1101/2024.10.12.24315151; this version posted October 15, 2024. The copyright holder for this preprint (which was not certified by peer review) is the author/funder, who has granted medRxiv a license to display the preprint in perpetuity.
It is made available under a CC-BY 4.0 International license .

Supplementary Figures

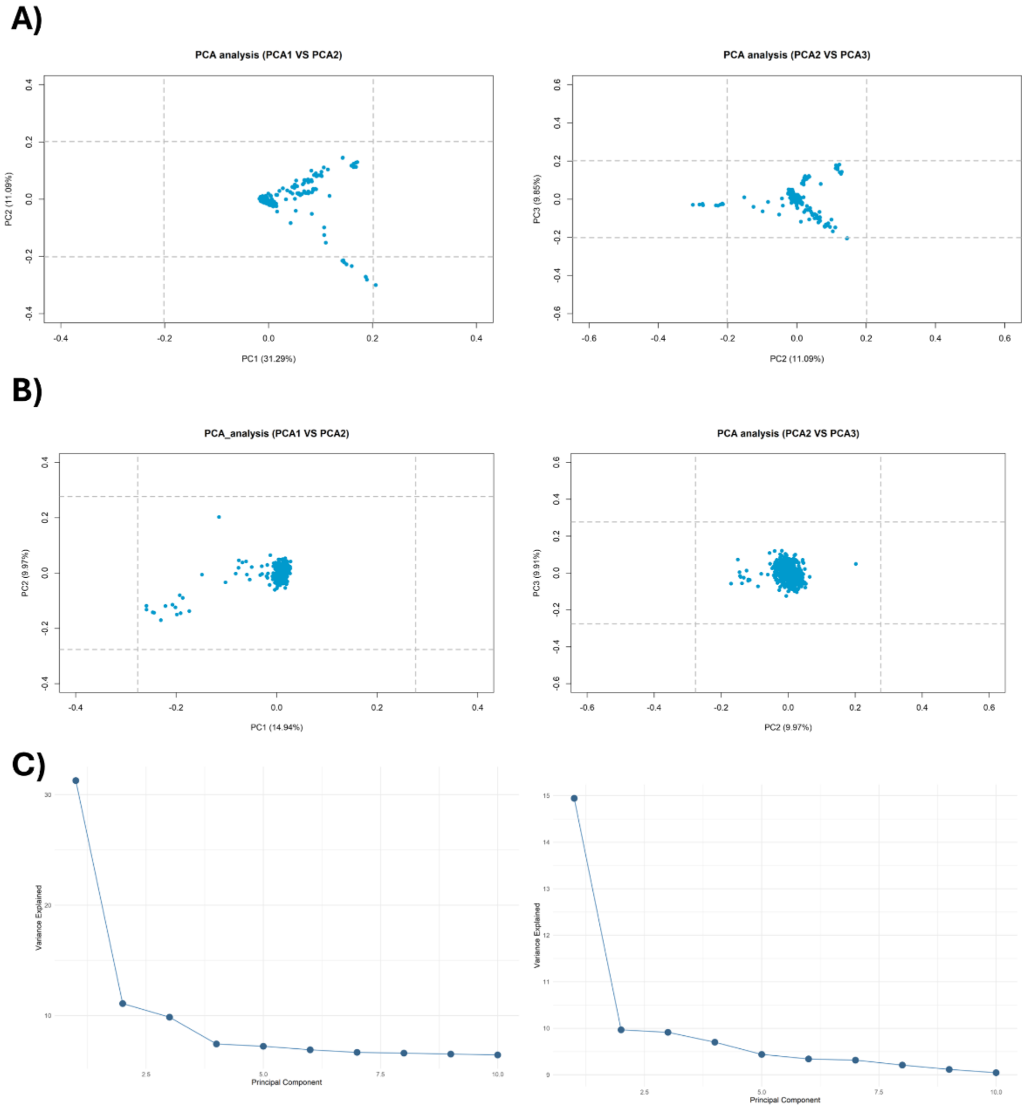

Supplementary Figure 1. Principal component analysis. A) Plot of the first two (left) and the second and third (right) principal components of genetic variation of IPF patients in the PFFPR. B) Plot of the first two (left) and the second and third (right) principal components of genetic variation of IPF patient in PROFILE. C) Proportion of variance explained by each PC (PFFPR on the right, and PROFILE on the left).

medRxiv preprint doi: https://doi.org/10.1101/2024.10.12.24315151; this version posted October 15, 2024. The copyright holder for this preprint (which was not certified by peer review) is the author/funder, who has granted medRxiv a license to display the preprint in perpetuity.
It is made available under a CC-BY 4.0 International license .

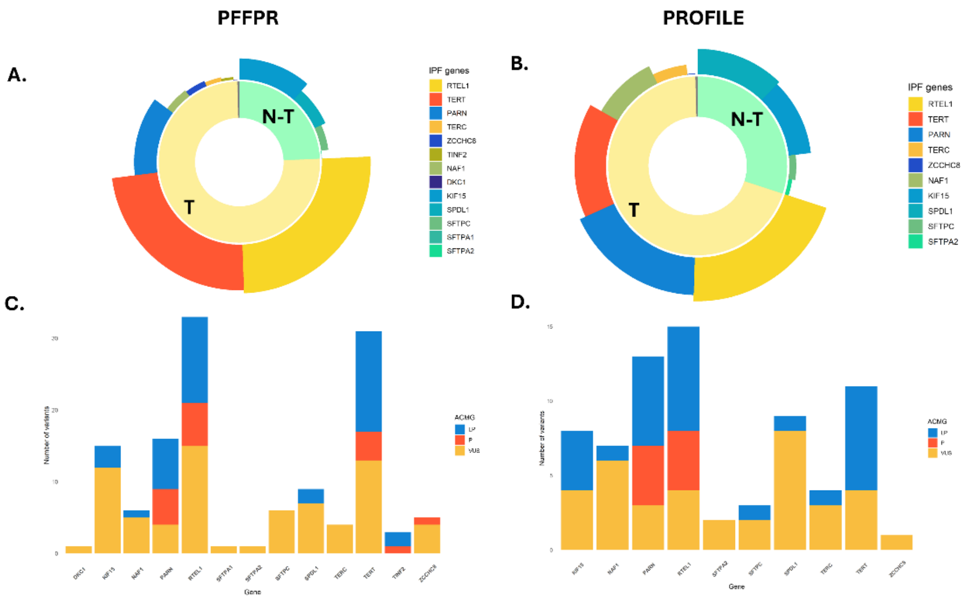

Supplementary Figure 2. Distribution of qualifying variants (QV) in monogenic adult-onset pulmonary fibrosis (PF) genes in the PFFPF and PROFILE cohorts. A) Total QVs in monogenic adult-onset PF genes in the PFFPR. B) Total QVs in monogenic adult-onset PF genes in the PROFILE cohort. C) Variants classified in P/LP/VUS per gene in the PFFPR. D) Variants classified in P/LP/VUS per gene in the PROFILE cohort. T: Telomere; N-T: Non telomere.

medRxiv preprint doi: https://doi.org/10.1101/2024.10.12.24315151; this version posted October 15, 2024. The copyright holder for this preprint (which was not certified by peer review) is the author/funder, who has granted medRxiv a license to display the preprint in perpetuity.
It is made available under a CC-BY 4.0 International license .

A.

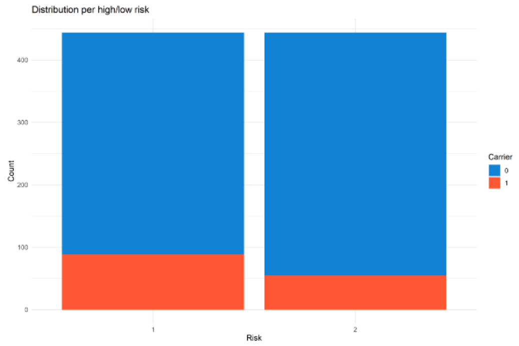

B.

	Low risk	High risk
OR (95% CI)	1.74 (1.20-2.53)	reference
p-value	3.57×10^{-3}	reference

Supplementary Figure 3. Association between prevalence of qualifying variants (QV) and PRS-IPF in the PFFPR. A) Distribution of carriers (1) and non-carriers (0) in low and high PRS-IPF. B) Risk of carrying a QV in patients with low polygenic risk in comparison with individuals with high polygenic risk. The odds ratio (OR) and the 95% confidence interval (CI) were estimated using logistic regression adjusted by age of diagnosis, sex, and the two main principal components.

medRxiv preprint doi: https://doi.org/10.1101/2024.10.12.24315151; this version posted October 15, 2024. The copyright holder for this preprint (which was not certified by peer review) is the author/funder, who has granted medRxiv a license to display the preprint in perpetuity.
It is made available under a CC-BY 4.0 International license .

A.

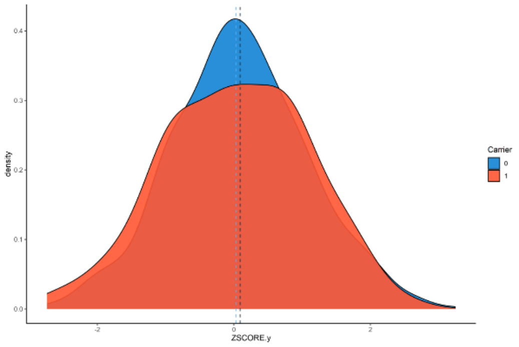

B.

	T1	T2	T3
**OR (95% CI)**	1.60 (1.01-2.54)	Reference	1.46 (0.92-2.32)
**p-value**	0.05	Reference	0.11

Supplementary Figure 4. Association between prevalence of qualifying variants (QV) and PRS-IPF (after excluding the *MUC5B* locus) in the PFFPR. A) Distribution of PRS-IPF in carriers (1) and non-carriers (0). Vertical dotted lines represent the mean value of the distribution. B) Risk of carrying a QV for patients with low polygenic risk (T1) and high polygenic risk (T3) compared to those in the middle tertile. The odds ratios (OR) and the 95% confidence intervals (CI) were estimated using logistic regression adjusted for age of diagnosis, sex, and the two main principal components.

medRxiv preprint doi: https://doi.org/10.1101/2024.10.12.24315151; this version posted October 15, 2024. The copyright holder for this preprint (which was not certified by peer review) is the author/funder, who has granted medRxiv a license to display the preprint in perpetuity.
It is made available under a CC-BY 4.0 International license .

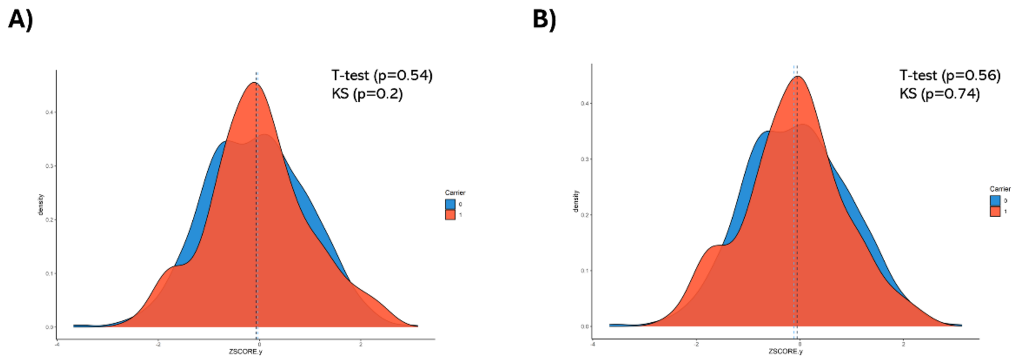

Supplementary Figure 5. Association between the prevalence of qualifying variants (QV) and PRS-TL in the PFFPR. Distribution of PRS-TL in carriers (1) and non-carriers (0). Vertical dotted lines represent the mean value of the distribution A) Carriers (1) and non-carriers (0) in telomere and non-telomere genes. B) Carriers (1) and non-carriers (0) in telomere genes. T-test: Student's t-test; KS: Kolmogorov-Smirnov test.

medRxiv preprint doi: https://doi.org/10.1101/2024.10.12.24315151; this version posted October 15, 2024. The copyright holder for this preprint (which was not certified by peer review) is the author/funder, who has granted medRxiv a license to display the preprint in perpetuity.
It is made available under a CC-BY 4.0 International license .

A)

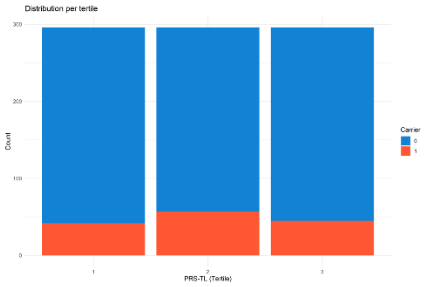

C)

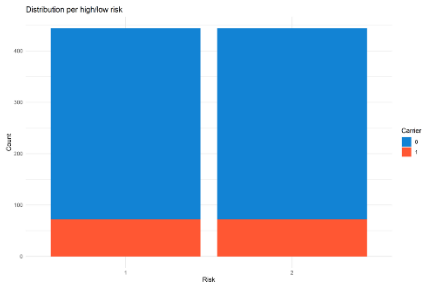

B)

	T1	T2	T3
OR (95% CI)	0.71 (0.46-1.12)	Reference	0.81 (0.53-1.26)
p-value	0.14	Reference	0.36

D)

	Low risk	High risk
OR (95% CI)	Reference	1.01 (0.70-1.46)
p-value	Reference	0.95

Supplementary Figure 6. Association between prevalence of qualifying variants (QV) in telomere and non-telomere genes and PRS-TL in the PFFPR. A) Distribution of carriers (1) and non-carriers (0) in PRS-TL tertiles. B) Risk of carrying a QV for individuals with low polygenic risk (T1) and high polygenic risk (T3) compared to those in the middle tertile. C) Distribution of carriers (1) and non-carriers (0) in high and low PRS-TL. D) Risk of carrying a QV in patients with high polygenic risk in comparison with patients with low polygenic risk. The odds ratios (OR) and the 95% confidence intervals (CI) were estimated using logistic regression adjusted by age of diagnosis, sex, and the two main principal components.

medRxiv preprint doi: https://doi.org/10.1101/2024.10.12.24315151; this version posted October 15, 2024. The copyright holder for this preprint (which was not certified by peer review) is the author/funder, who has granted medRxiv a license to display the preprint in perpetuity.
It is made available under a CC-BY 4.0 International license .

A)

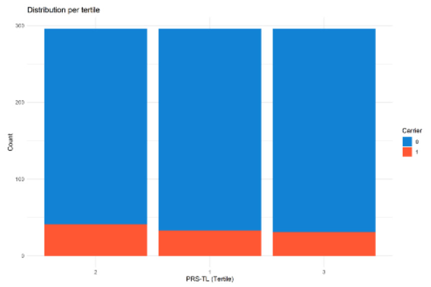

C)

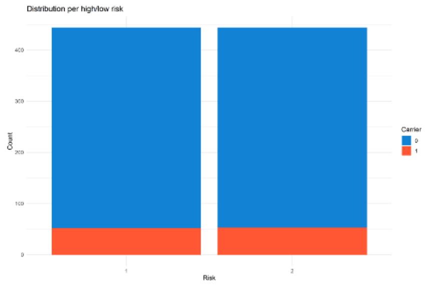

B)

	T1	T2	T3
**OR (95% CI)**	0.81 (0.49-1.34)	Reference	0.81 (0.49-1.35)
**p-value**	0.41	Reference	0.42

D)

	Low risk	High risk
**OR (95% CI)**	Reference	1.04 (0.69-1.59)
**p-value**	Reference	0.83

Supplementary Figure 7. Association between prevalence of qualifying variants (QV) in telomere genes and PRS-TL in the PFFPR. A) Distribution of carriers (1) and non-carriers (0) in PRS-TL tertiles. B) Risk of carrying a QV for individuals with low polygenic risk (T1) and high polygenic risk (T3) compared to those in the middle tertile. C) Distribution of carriers (1) and non-carriers (0) in high and low PRS-TL. D) Risk of carrying a QV in individuals with high polygenic risk in comparison with individuals with low polygenic risk. The odds ratios (OR) and the 95% confidence intervals (CI) were estimated using logistic regression adjusted by age of diagnosis, sex, and the two main principal components.

medRxiv preprint doi: https://doi.org/10.1101/2024.10.12.24315151; this version posted October 15, 2024. The copyright holder for this preprint (which was not certified by peer review) is the author/funder, who has granted medRxiv a license to display the preprint in perpetuity.
It is made available under a CC-BY 4.0 International license .

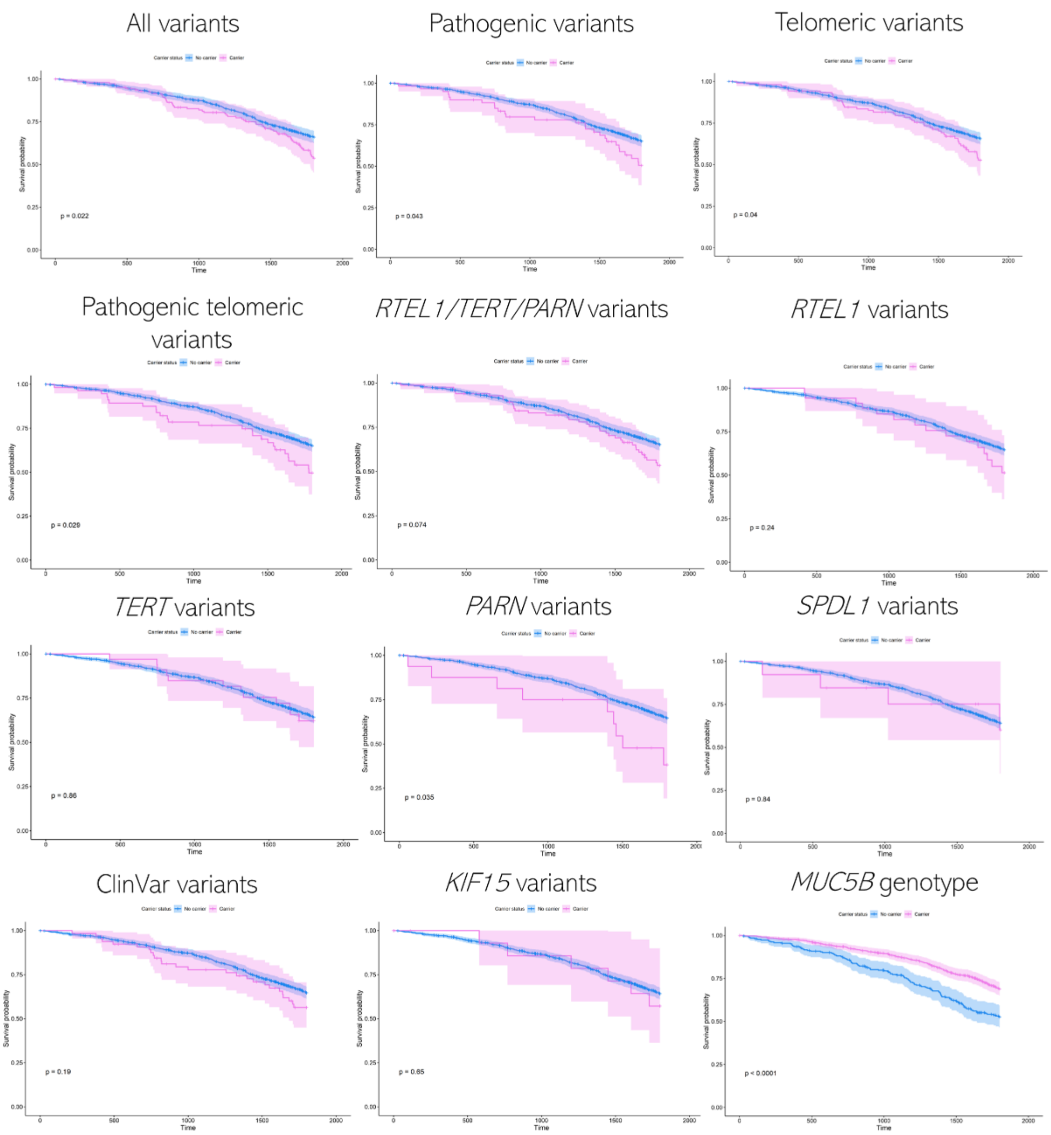

Supplementary Figure 8. Kaplan-Meier survival analysis for qualifying variants (QV) (per gene and group of genes) and the *MUC5B* risk allele in the PFFPR. p-values for the log-rank test are shown.

medRxiv preprint doi: https://doi.org/10.1101/2024.10.12.24315151; this version posted October 15, 2024. The copyright holder for this preprint (which was not certified by peer review) is the author/funder, who has granted medRxiv a license to display the preprint in perpetuity.
It is made available under a CC-BY 4.0 International license .

Variable	Carrier	Non carrier	HR, 95% CI, p-value
All variants	128	744	1.46 (1.04-2.03, p=0.027)
Pathogenic variants	48	824	1.46 (0.88-2.43, p=0.140)
Telomeric variants	89	783	1.37 (0.93-2.00, p=0.107)
Pathogenic telomeric variants	44	828	1.50 (0.89-2.52, p=0.128)
Clinvar	58	814	1.22 (0.76-1.96, p=0.416)
TERT/RTEL1 variants	68	804	1.30 (0.84-2.02, p=0.243)

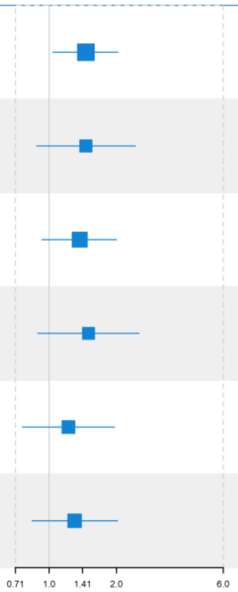

Supplementary Figure 9. Qualifying variants (QV) effect on survival in the PFFPR (excluding carriers of QV within *PARN*). All analysis were performed using Cox regression models adjusted for sex, age of diagnosis, the two main principal components, *MUC5B* risk allele, smoking history, forced vital capacity (FVC) % predicted, and diffusing capacity for carbon monoxide (DLCO) % predicted. The X-axis shows Hazard-ratios (HR); the grey line corresponds to the HR=1.0. The boxes correspond to adjusted HR and horizontal lines correspond to 95% confidence intervals (CI).

medRxiv preprint doi: https://doi.org/10.1101/2024.10.12.24315151; this version posted October 15, 2024. The copyright holder for this preprint (which was not certified by peer review) is the author/funder, who has granted medRxiv a license to display the preprint in perpetuity.
It is made available under a CC-BY 4.0 International license .

Variable	Carrier	Non carrier	HR, 95% CI, p-value
All rare variants	144	744	1.53 (1.12-2.10, p=7.3E-3
rare ensemble	64	824	1.34 (0.86-2.10, p=0.192)
rare PTV	28	860	1.36 (0.71-2.61, p=0.351)
semi ensemble	75	813	1.66 (1.11-2.50, p=0.014)
semi PTV	29	859	1.51 (0.81-2.82, p=0.194)
ultra ensemble	30	858	1.72 (0.98-3.02, p=0.057)
ultra PTV	13	875	2.44 (1.14-5.24, p=0.022)

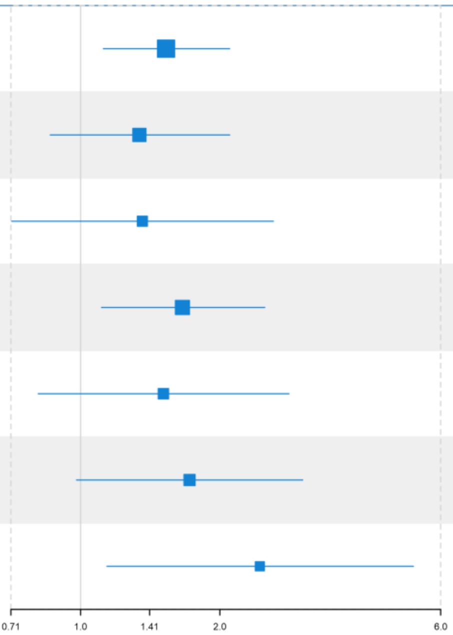

Supplementary Figure 10. **Alternative qualifying variants (QV) classifications and effects on survival in the PFFPR**. All analysis were performed using Cox regression models adjusted for sex, age of diagnosis, the two main principal components, *MUC5B* risk allele, smoking history, forced vital capacity (FVC) % predicted, and diffusing capacity for carbon monoxide (DLCO) % predicted. The X-axis shows Hazard-ratios (HR); the grey line corresponds to the HR=1.0. The boxes correspond to adjusted HR and horizontal lines correspond to 95% confidence intervals (CI).

medRxiv preprint doi: https://doi.org/10.1101/2024.10.12.24315151; this version posted October 15, 2024. The copyright holder for this preprint (which was not certified by peer review) is the author/funder, who has granted medRxiv a license to display the preprint in perpetuity.
It is made available under a CC-BY 4.0 International license .

A)

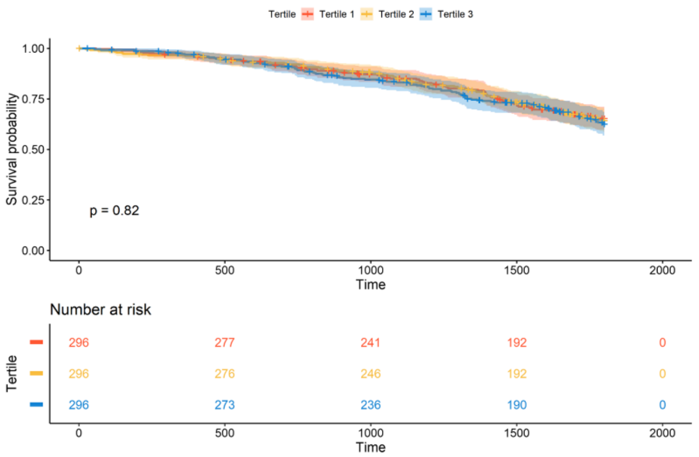

B)

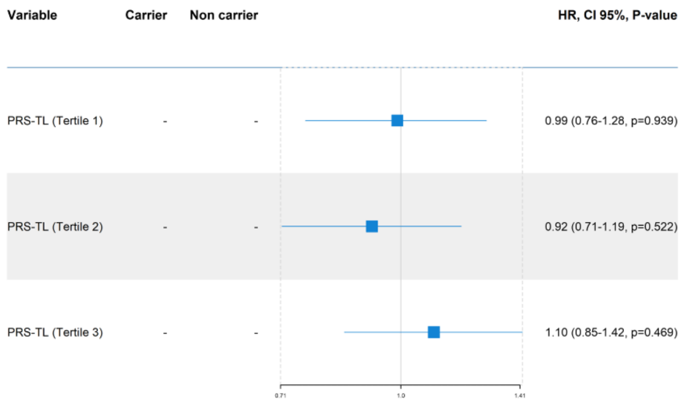

Supplementary Figure 11. Association between PRS-TL tertiles and survival in the PFFPR. A) Kaplan-Meier survival analysis for PRS-TL tertiles (p-value for the log-rank test is shown). B) PRS-TL effect on survival. The analysis was performed using Cox regression models adjusted for sex, age of diagnosis, the two main principal components, smoking history, forced vital capacity (FVC) % predicted, and diffusing capacity for carbon monoxide (DLCO) % predicted. The X-axis shows Hazard-ratios (HR); the grey line corresponds to the HR=1.0. The boxes correspond to adjusted HR and horizontal lines correspond to 95% confidence intervals (CI).

medRxiv preprint doi: https://doi.org/10.1101/2024.10.12.24315151; this version posted October 15, 2024. The copyright holder for this preprint (which was not certified by peer review) is the author/funder, who has granted medRxiv a license to display the preprint in perpetuity.
It is made available under a CC-BY 4.0 International license .

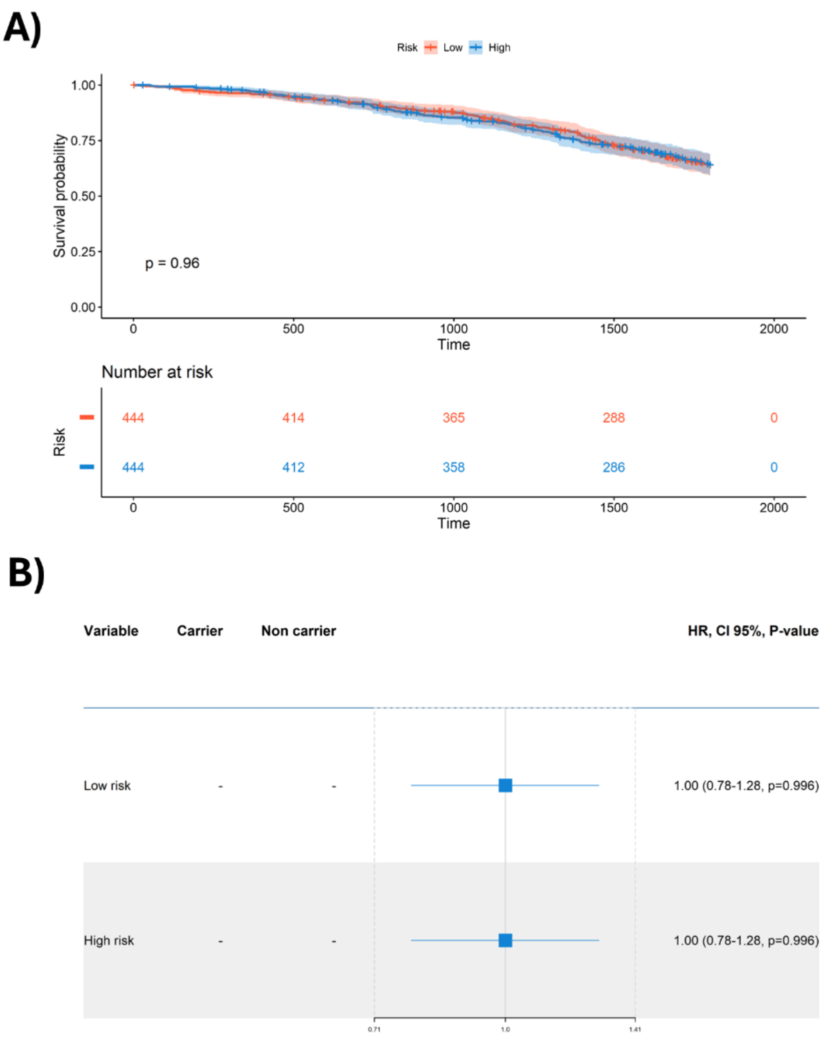

Supplementary Figure 12. Association between high and low PRS-TL and survival in the PFFPR. A) Kaplan-Meier survival analysis for high/low risk PRS-TL (p-value for the log-rank test is shown). B) PRS-TL effect on survival. The analysis was performed using Cox regression models adjusted for sex, age of diagnosis, the two main principal components, smoking history, forced vital capacity (FVC) % predicted, and diffusing capacity for carbon monoxide (DLCO) % predicted. The X-axis shows Hazard-ratios (HR); the grey line corresponds to the HR=1.0. The boxes correspond to adjusted HR and horizontal lines correspond to 95% confidence intervals (CI).

medRxiv preprint doi: https://doi.org/10.1101/2024.10.12.24315151; this version posted October 15, 2024. The copyright holder for this preprint (which was not certified by peer review) is the author/funder, who has granted medRxiv a license to display the preprint in perpetuity.
It is made available under a CC-BY 4.0 International license .

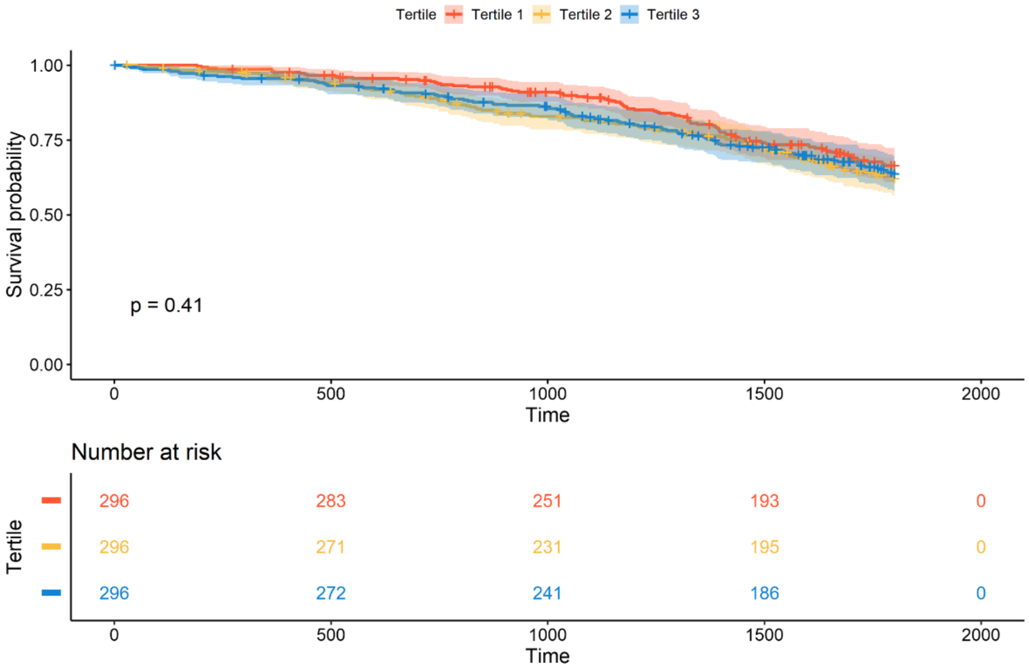

Supplementary Figure 13. Association of PRS-IPF (after excluding the *MUC5B* locus) and survival in the PFFPR. Kaplan-Meier analysis showing p-values for the log-rank test.

medRxiv preprint doi: https://doi.org/10.1101/2024.10.12.24315151; this version posted October 15, 2024. The copyright holder for this preprint (which was not certified by peer review) is the author/funder, who has granted medRxiv a license to display the preprint in perpetuity.
It is made available under a CC-BY 4.0 International license .

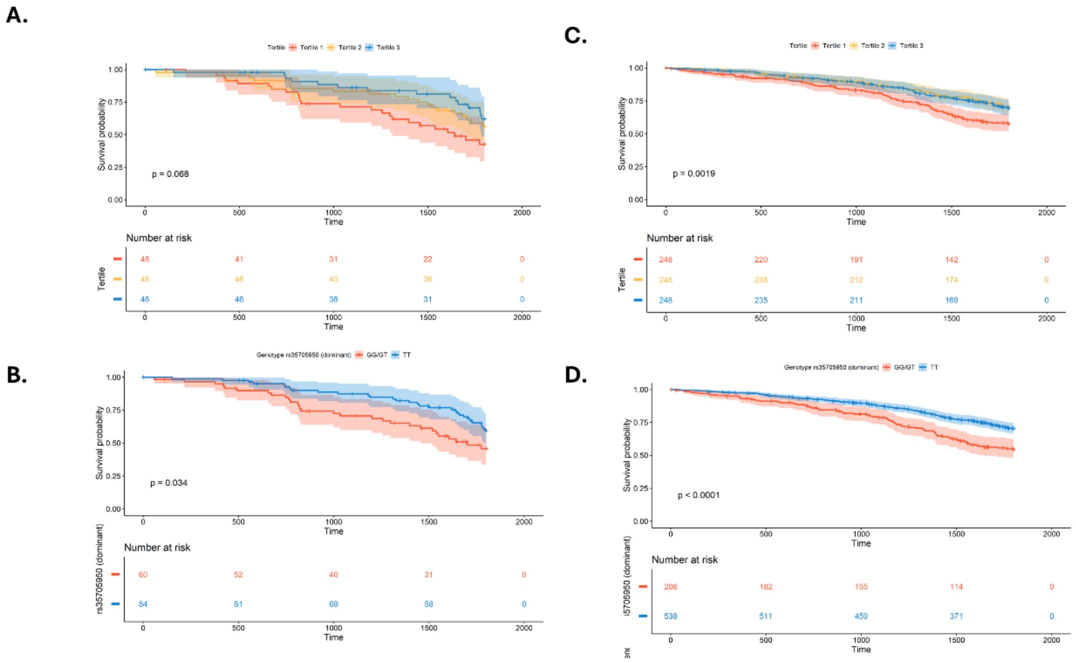

Supplementary Figure 14. Associations between PRS-IPF and *MUC5B* rs35705950 genotypes with survival among carriers and non-carriers of qualifying variants (QV) in the PFFPR. A) Association between PRS-IPF and survival in carriers. B) Association between PRS-IPF and survival in non-carriers. C) Association between *MUC5B* rs35705950 genotypes and survival in carriers. D) Association between *MUC5B* rs35705950 genotypes and survival in non-carriers. Kaplan-Meier analysis, showing p-values for the log-rank test.

medRxiv preprint doi: https://doi.org/10.1101/2024.10.12.24315151; this version posted October 15, 2024. The copyright holder for this preprint (which was not certified by peer review) is the author/funder, who has granted medRxiv a license to display the preprint in perpetuity.
It is made available under a CC-BY 4.0 International license .

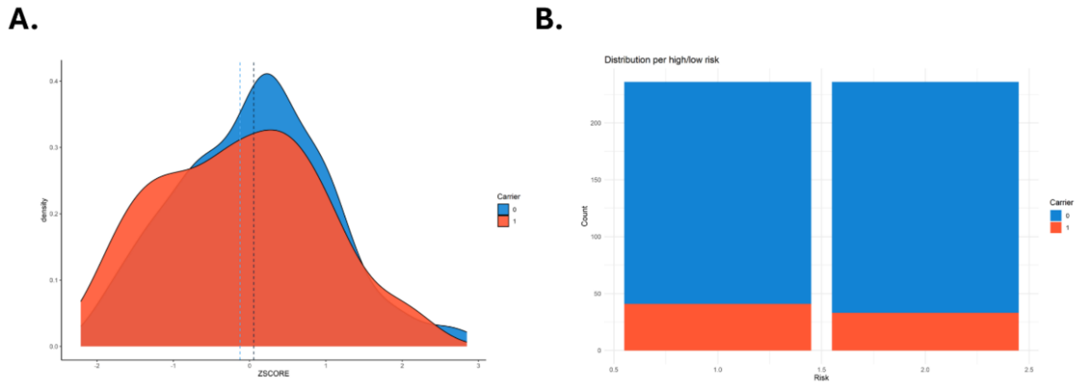

Supplementary Figure 15. Association between prevalence of qualifying variants (QV) and PRS-IPF in PROFILE. A) Distribution of PRS-IPF in carriers (1) and non-carriers (0). Vertical dotted lines represent the mean value of the distribution. B) Distribution of carriers (1) and non-carriers (0) in high and low PRS-IPF.

medRxiv preprint doi: https://doi.org/10.1101/2024.10.12.24315151; this version posted October 15, 2024. The copyright holder for this preprint (which was not certified by peer review) is the author/funder, who has granted medRxiv a license to display the preprint in perpetuity.
It is made available under a CC-BY 4.0 International license .

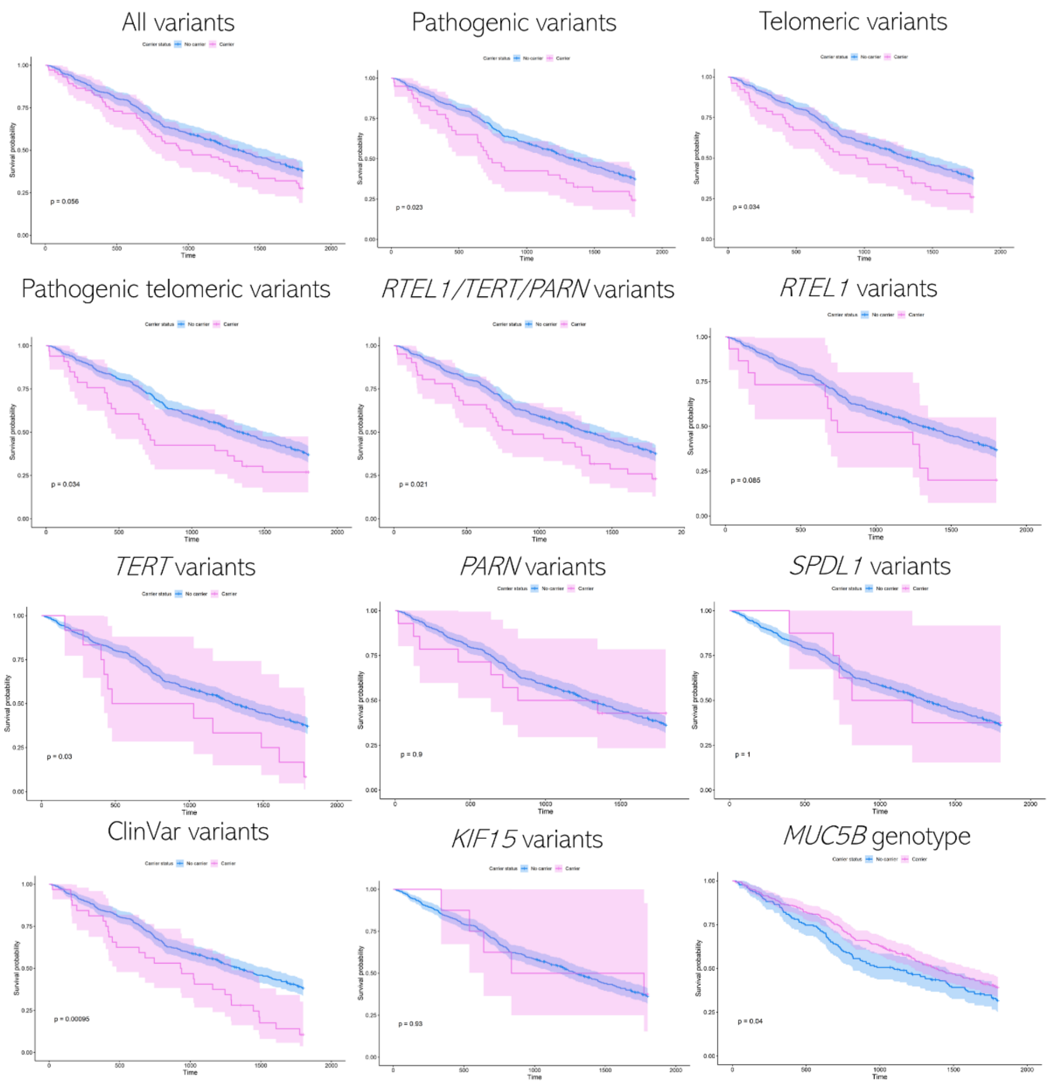

Supplementary Figure 16. Kaplan-Meier survival analysis for qualifying variants (QV) (per gene and group PF genes) and the *MUC5B* risk allele in PROFILE. p-values for the log-rank test are shown.

medRxiv preprint doi: https://doi.org/10.1101/2024.10.12.24315151; this version posted October 15, 2024. The copyright holder for this preprint (which was not certified by peer review) is the author/funder, who has granted medRxiv a license to display the preprint in perpetuity.
It is made available under a CC-BY 4.0 International license .

Supplementary references

1. Wang, B. R. *et al.* The Pulmonary Fibrosis Foundation Patient Registry. Rationale, Design, and Methods. *Ann Am Thorac Soc* **17**, 1620–1628 (2020).

2. Maher, T. M. PROFILEing idiopathic pulmonary fibrosis: rethinking biomarker discovery. *European Respiratory Review* **22**, 148–152 (2013).

3. Maher, T. M. *et al.* An epithelial biomarker signature for idiopathic pulmonary fibrosis: an analysis from the multicentre PROFILE cohort study. *The Lancet Respiratory Medicine* **5**, 946–955 (2017).

4. Chang, C. C. *et al.* Second-generation PLINK: rising to the challenge of larger and richer datasets. *GigaScience* **4**, s13742-015-0047–8 (2015).

5. Pedersen, B. S. *et al.* Somalier: rapid relatedness estimation for cancer and germline studies using efficient genome sketches. *Genome Medicine* **12**, 62 (2020).

6. Manichaikul, A. *et al.* Robust relationship inference in genome-wide association studies. *Bioinformatics* **26**, 2867–2873 (2010).

7. Lu, W. *et al.* CHARR efficiently estimates contamination from DNA sequencing data. *The American Journal of Human Genetics* **110**, 2068–2076 (2023).
